# Internet addiction, depressive symptoms, and anxiety symptoms are associated with the risk of eating disorders among university students in Bangladesh

**DOI:** 10.1038/s41598-023-47101-z

**Published:** 2023-11-22

**Authors:** Md. Hasan Al Banna, Shammy Akter, Humayun Kabir, Keith Brazendale, Mst. Sadia Sultana, Najim Z. Alshahrani, Bright Opoku Ahinkorah, Tarif Salihu, Bably Sabina Azhar, Md. Nazmul Hassan

**Affiliations:** 1 Department of Food Microbiology, Faculty of Nutrition and Food Science, Patuakhali Science and Technology University, Patuakhali, 8602 Bangladesh; 2 Nutrition Initiative, Kushtia, Bangladesh; 3 Department of Applied Nutrition and Food Technology, Faculty of Biological Sciences, Islamic University, Kushtia, Bangladesh; 4 Department of Health Research Methods, Evidence and Impact, McMaster University, Hamilton, ON Canada; 5 Department of Health Sciences, University of Central Florida, Orlando, USA; 6 Department of Public Health and Informatics, Jahangirnagar University, Savar, Dhaka, Bangladesh; 7 Department of Family and Community Medicine, Faculty of Medicine, University of Jeddah, Jeddah, Saudi Arabia; 8 School of Public Health, University of Technology Sydney, Sydney, NSW Australia; 9 Department of Population and Health, University of Cape Coast, Cape Coast, Ghana; 10 Department of Environmental Sanitation, Faculty of Nutrition and Food Science, Patuakhali Science and Technology University, Patuakhali, 8602 Bangladesh

**Keywords:** Medical research, Risk factors

## Abstract

The risk of developing an eating disorder among university students is higher than the general population in Bangladesh. Since psychiatric disorders (such as depression and anxiety) and addictive behaviors (e.g., internet addiction) predominantly exist among university students in the country, these may increase their vulnerability to developing an eating disorder. The association of internet addiction, depression, and anxiety with the risk of eating disorders among Bangladeshi university students is relatively unknown; therefore, this study investigates the association. This study was a cross-sectional design. Students (N = 700) from two public universities in Bangladesh completed the Patient Health Questionnaire (PHQ-9) scale, the Generalized Anxiety Disorder (GAD-7) tool, and Orman's Internet Addiction Survey (OIAS) to measure exposure variables. Eating Attitudes Test-26 (EAT-26) assessed the outcome variable. Multivariable logistic regression analysis showed that internet addiction [adjusted odds ratio (aOR) for moderate addiction = 2.15 and severe addiction = 3.95], depressive (aOR 3.04), and anxiety (aOR 2.06) symptoms were associated with an increased risk of eating disorder among study participants. Future longitudinal studies on university students are recommended to gain a better understanding about the causal factors of eating disorder to support intervention initiatives and strategies by public health practitioners and policy experts.

## Introduction

### Significance of the university-aged period

The university-aged period (18–26 years), which falls into an emerging adulthood stratum of lifespan, is often considered a key developmental phase of life^1^. University students (i.e., young adults) go through this transition era where they leave the family home to pursue higher education, and their way of life changes due to academic obligations, growing flexibility and independence, and reform of peer relationships. Studies have shown that university students are at risk of various mental health conditions (e.g., depression, anxiety, etc.), addictive behaviors (such as internet addiction), unhealthy dietary habits, and eating disorders^2–5^.

### Prevalence and consequences of eating disorders

Eating disorders (EDs) are abnormal eating behaviors that impair social and psychological functioning^6^. EDs have been associated with various long-term physical consequences and a high risk for comorbidity and mortality, with the highest proportion of cause-specific death among psychiatric disorders^7,8^. Globally, approximately 42 million people suffer from different forms of EDs (e.g., anorexia and bulimia); however, people with EDs tend to not seek medical help^9,10^. Thus, early screening and diagnosis of individuals who are at risk of EDs are crucial to providing treatment and reducing disease burden.

Evidence shows that university or college students have a higher prevalence of EDs compared to the general population^11^, which can adversely impact students’ physical and mental health, lower academic functioning and functional impairment^12^. A recent systematic review and meta-analysis estimated that the prevalence of screen-based disordered eating behavior among university students, globally, was 20%^2^. In recent years, the risk of EDs appears to have increased among university students in Asian countries^13,14^, particularly in Bangladesh, with estimates ranging between 20.4 and 37.6%^15,16^. Previous studies conducted among students of public universities in Bangladesh reported that the prevalence of ED risk ranges between 20.4 and 23.0%^15,17^. A recent study undertaken among female Bangladeshi university students reported that approximately 37% of them were at risk of developing an ED^18^. Another previous study of Bangladeshi undergraduate university students revealed that the prevalence of developing an ED risk was 37.6%^16^. The increasing trends of disordered eating behaviors in Asian regions may be due to economic expansion, social transformation in gender responsibilities and conventional family constructions, greater exposure to the Western tradition and standards of beauty, and increased access to junk and fast foods^19^.

### Factors associated with eating disorder risk

The etiology of EDs is complex with a wide range of sociocultural, psychological, and biological influences^20^. Previous studies have identified several factors associated with ED risk or disordered eating attitudes in different settings, such as: (i) sociodemographic and cultural factors: age, female sex, married status, exposure to cultural transition, globalization, urbanization and lack of religiosity^21–24^; (ii) body-related factors: body mass index (BMI), body shape concern, desire for thinness, and cosmetic surgery^24–26^; and (iii) psychiatric and behavioral factors: depression, anxiety and pathological internet use^27–29^.

### Knowledge gap regarding eating disorder risk among university students in Bangladesh

In Bangladesh, a key area of public health concern is common mental health problems including depression and anxiety, as well as internet use/internet addiction among university students^4,5^. A meta-analysis by Puccio et al.^28^ concluded that eating pathology and depression are concurrent risk factors for each other, with other studies showing problematic internet use is a predictor of EDs in students^30^; however, contradictory findings have also been reported. One study of university students in the Association of Southeast Asian Nations (i.e., ASEAN refers to Indonesia, Malaysia, Myanmar, Thailand and Vietnam) found that depression symptoms and pathological internet use were significantly associated with ED risk^13^. Although students are one of the most susceptible groups to developing ED, in Bangladesh, few studies have investigated ED risk among university students and additional sociodemographic, health/lifestyle, and/or behavioral factors associated with EDs risk^15,16,18^.

Since psychiatric disorders and addictive behaviors predominantly exist among Bangladeshi university students, these could make them more vulnerable to disordered eating habits or ED risk. Thus, in this study, we included university students’ socio-demographic characteristics, internet addiction, depression, and anxiety symptoms to observe the estimated adjusted effect of these factors on ED risk. The main objective of the present study was to assess the association between mental health conditions (such as depression and anxiety) and addictive behaviors (e.g., internet addiction) with the risk of ED among university students in Bangladesh. Initial hypotheses were female gender, internet addiction, and depressive and anxiety symptoms would be associated with the risk of ED in Bangladeshi university students.

## Materials and methods

### Study design and sample source

A cross-sectional study was undertaken among students sampled from two conveniently selected public universities in Bangladesh. The following eligibility criteria were set up to enroll a study participant: (i) being at least 18 years old (i.e., adults), (ii) being a Bangladeshi citizen by birth, and (iii) being a current student. Moreover, participants who had any prior history of a clinically diagnosed ED and medical conditions such as diabetes and hypertension were excluded from the study due to the potential for research outcomes to be overestimated or underestimated. The study was conducted between May and October 2022.

### Sample size estimation and sampling

A minimum required sample was estimated using a single population proportion test. The following assumptions were factored in: (i) a 23.0% prevalence of ED risk among Bangladeshi public university students was used (p = 0.23)^17^, (ii) 95% confidence (Z = 1.96), and (iii) 5% margin of error (d = 0.05). The calculation formula was as follows:

$$\text{Minimum \, sample \, size, n = }\frac{ {\text{z}}^{2} \times {\text{p}} \times (1-{\text{p}})}{{\text{d}}^{2}}\text{=}\frac{{(1.96)}^{2}{ \times 0.23 \times (1-0.23)}}{{(0.05)}^{2}} = 272.14 \approx 272.$$

A 10% non-response rate was also considered, resulting in an optimal sample size of 300. To increase the external validity and generalizability of the study, we targeted to include more participants than our calculated sample size^31^. Thus, a total of 700 participants constituted the final sample size in this study.

A simple random technique was applied to recruit and enroll participants. Three field investigators (i.e., interviewers) randomly visited different areas on the university campuses where students gather together to spend their leisure time such as the cafeteria, tea stall, central field, teacher student center (TSC), etc. There are no restrictions on accessing public university campuses in Bangladesh, thus, the field investigators randomly approached students on the campuses, clarified the study’s purpose to them, and assessed study eligibility criteria. Almost equal proportions of participants were recruited from each of the two universities (i.e., 345 vs. 355 participants).

### Ethics and study procedure

Prior to data collection study procedures and protocols related to human subjects research was approved by the Research Ethical Committee (REC) of Department of Environmental Sanitation, Patuakhali Science and Technology University, Bangladesh [approval number: ENS:09/04/2022:06]. All methods were carried out in accordance with the relevant guidelines and regulations. Interviewers, who were trained by the principal investigator, collected data through a person-to-person interview using a paper-based questionnaire comprising of the study variables measures. Before beginning the questionnaire, written informed consent was obtained and the study's purpose was explained to the participants, and it was made clear that study participation was voluntary. Participants’ de-identifying information and the confidentiality of their data were strictly maintained. The interviewer read each question on the questionnaire with all possible response options to the participant and completed it in-person.

### Measures

#### Sociodemographic, lifestyle and behavioral information

Gender, educational background, study level, academic performance (CGPA < 3.0 = poor vs. CGPA > 3.0 = good), marital status, residence, monthly family income, and family size were included as participants’ socio-demographic variables. Lifestyle and behavior-related information included tobacco smoking status (yes vs. no), physical activity level (physically inactive, moderate activity or regular activity), self-perceived BMI status (underweight, normal body weight or overweight/obesity) and sleep duration (long: average 9 ≥ h sleep/night in the past 4 weeks), normal: 7 and 8 h or short: ≤ 6 h)^32^.

#### Depressive symptoms

To assess depressive symptoms, we used the Patient Health Questionnaire (PHQ-9) scale which contains nine individual items^33^. For the PHQ-9 scale, the result of each item was coded by “Not at all” (0) to “Nearly every day” (3). Participants were asked how often in the past two weeks they had been bothered by the nine problems such as "Little interest or pleasure in doing things". Two categories were created from the total score. The cut-off point was 10 vs. less, with scores of less than 10 being classified as not having depressive symptoms and scores of 10 or more as having depressive symptoms^34^. The internal consistency of the scale was adequate (Cronbach's α = 0.74). This scale was previously used in epidemiological studies in Bangladesh^32,35^, and is valid and reliable for screening depressive symptoms among university students in Bangladesh^36^.

#### Anxiety symptoms

The severity of study participants' anxiety symptoms was evaluated using the Generalized Anxiety Disorder (GAD-7) tool. In epidemiological studies, the GAD-7 is a useful and reliable tool for detecting anxiety symptoms^35,37^. The GAD-7 scale consists of seven items, each of which is based on a four-point Likert scale, with 0 (Never) to 3 (Nearly every day). For instance, participants were asked how they responded to the following problems (over the past 2 weeks): such as, “feeling nervous, anxious, or on edge”, “worrying too much about different things”, etc. A higher number denotes more severe anxiety symptoms, with the total score ranging from 0 to 21. Those scoring ≥ 10 were considered to have anxiety symptoms in this study^35,38^. This scale is validated in Bangladeshi university students, with high internal consistency and good convergent validity^39^. In the present study, an acceptable level of reliability of the scale was found (Cronbach’s α = 0.71).

#### Internet addiction

The level of internet addiction was calculated using 9-items (Yes vs. No) Orman's Internet Addiction Survey (OIAS)^40^. For example, the first question was as follows: “Do you have problems controlling your impulse to connect to internet?” The calculation consists of adding up the total number of "Yes" responses to the test's nine questions. A score of three or lower would indicate a complete lack of reliance or addiction, a score of four to six points would indicate a moderate online addiction, and a score of seven or more would indicate serious internet addiction. A previous study also recommended the use of OIAS to define internet addiction^41^. A previous Bangladeshi study also used the OIAS to assess students’ level of internet addiction^42^. The reliability of this scale was tested by Kuder-Richardson Formula 20 (KR-20) and found acceptable internal consistency in the present sample (KR-20 = 0.70).

#### Eating disorder risk (outcome variable)

The risk of ED was assessed using the validated self-reported Eating Attitude Test-26 (EAT-26) questionnaire, which was a six-point Likert scale from ‘always’ to ‘never’^43^. The participants were given a response for each of the 26 statements, including “I am terrified about being overweight”, “I avoid eating when I am hungry”, etc. For scoring purposes, a point of 3, 2 and 1 was assigned for ‘always’, ‘usually’ and ‘often’, respectively, and 0 for ‘sometimes’, ‘rarely’ and ‘never’ (inverse scoring for item-26). A score of ≥ 20 was categorized as ‘at risk’, and < 20 as ‘no risk’, for ED (score range 0 to 78)^16,17^. Cronbach’s α of this scale was 0.83, which indicates an excellent level of internal consistency.

### Statistical approach

Data were analyzed by STATA (BE version 16.0, StataCorp, College Station, TX, USA) and SPSS (Windows version 23.0). Enumerative statistics, including frequency, percentage, mean and standard deviation (SD) were computed. A chi-square test was performed to show the distribution of the outcome variable across the independent variables. A bivariate and multivariable logistic regression model was fitted to identify the factors associated with ED risk. The nature of the variables derived from the data met all the key assumptions of a binary logistic regression. First, the dependent variable of this study was dichotomous (i.e., at-risk or no risk for ED). That is, it is either present or absent but never both at once. Second, since all our variables were categorical and binary, we confirmed using the proportional distribution of the categories in terms of their number and distribution across the outcome variable that there were no extreme outliers. Finally, before performing the adjusted regression model, all explanatory variables (16 variables) were tested for multicollinearity using variance inflation factor (VIF), and no multicollinearity was observed [mean VIF = 1.433, minimum VIF = 1.035, Maximum VIF = 3.123]. In the bivariate logistic regression model, the odds ratio with the corresponding confidence interval (CI at 95% level) was computed for all variables. A backward binary logistic regression method was used to construct the best model to explain the data, where the model starts with a full model and gradually drops the least significant variables from the model. The adjusted model was fitted based on the Hosmer and Lemeshow goodness of fit test (Chi-square = 9.466, df = 8, p = 0.305). The threshold for statistical significance was set at < 0.05.

## Results

### Sample characteristics

The study included 700 university students between the age range of 18 to 27 years (mean age 23.11 years, SD 1.77), with males making up more than half (52.0%) of the sample. The response rate of this study was 91.02%. Based on the PHQ-9 scale (mean score 9.35, SD 5.71), around 39.1% of the participants were identified as having depressive symptoms. The average score of the GAD-7 scale was 8.42 (SD 4.62) and a quarter (25.9%) of the participants were found to have anxiety symptoms. According to the OIAS scale (mean score 5.10, SD 3.14), nearly two-thirds (66.6%) of the participants had moderate to severe levels of internet addiction (Table 1).

**Table 1:** Socio-demographic, health, lifestyle, and behavioral characteristics of study participants (N = 700).

Variables	Total; n (%)	Eating disorder risk		Chi-square statistic	
		Yes	No	Chi-square	P value
Gender				7.15 (1)	**0.008**
Male	364 (52.0)	95 (26.1)	269 (73.9)		
Female	336 (48.0)	119 (35.4)	217 (64.6)		
Age in years				15.88 (2)	** < 0.001**
18–20	96 (13.7)	24 (25.0)	72 (75.0)		
21–24	436 (62.3)	118 (27.1)	318 (72.9)		
25 and above	168 (24.0)	72 (42.9)	96 (57.1)		
Study major				8.89 (4)	0.064
Engineering	107 (15.3)	36 (33.6)	71 (66.4)		
Health science	181 (25.9)	44 (24.3)	137 (75.7)		
Biological science	203 (29.0)	67 (33.0)	136 (67.0)		
Social science	94 (13.4)	37 (39.4)	57 (60.6)		
Others	115 (16.4)	30 (26.1)	85 (73.9)		
Study level				21.47 (4)	** < 0.001**
1st year	168 (24.0)	43 (25.6)	125 (74.4)		
2nd year	173 (24.7)	51 (29.5)	122 (70.4)		
3rd year	138 (19.7)	32 (23.2)	106 (76.8)		
4th year	123 (17.6)	40 (32.5)	83 (67.5)		
Masters	98 (14.0)	48 (49.0)	50 (51.0)		
Academic performance				0.34 (1)	0.562
Poor (CGPA < 3.0)	296 (42.3)	87 (29.4)	209 (70.6)		
Good (CGPA > 3.0)	404 (57.7)	127 (31.4)	277 (68.6)		
Marital status				5.47 (1)	**0.019**
Single	590 (84.3)	170 (28.8)	420 (71.2)		
Married	110 (15.7)	44 (40.0)	66 (60.0)		
Residence				5.77 (2)	0.056
Urban	464 (66.3)	130 (28.0)	334 (72.0)		
Sub-urban	49 (7.0)	21 (42.9)	28 (57.1)		
Rural	187 (26.7)	63 (33.7)	124 (66.3)		
Family income (BDT, monthly)				8.79 (1)	**0.003**
≤ 35,000	360 (51.4)	92 (25.6)	268 (74.4)		
> 35,000	340 (48.6)	122 (35.9)	218 (64.1)		
Family size				0.08 (1)	0.782
Nuclear (< 5 members)	398 (56.9)	120 (30.2)	278 (69.8)		
Extended (> 5members)	302 (43.1)	94 (31.1)	208 (68.9)		
Tobacco smoking status				41.28 (1)	** < 0.001**
Yes	265 (37.9)	119 (44.9)	146 (55.1)		
No	435 (62.1)	95 (21.8)	340 (78.2)		
Physical activity level				0.14 (2)	0.934
Physically inactive	142 (20.3)	44 (31.0)	98 (69.0)		
Moderate activity	318 (45.4)	95 (29.9)	223 (70.1)		
Regular activity	240 (34.3)	75 (31.2)	165 (68.8)		
Sleep duration				2.19 (2)	0.334
Short	342 (48.9)	101 (29.5)	241 (70.5)		
Normal	165 (23.6)	58 (35.2)	107 (64.8)		
Long	193 (27.6)	55 (28.5)	138 (71.5)		
Self-reported BMI Status				26.47 (2)	** < 0.001**
Underweight	88 (12.6)	13 (14.8)	75 (85.2)		
Normal weight	418 (59.7)	117 (28.0)	301 (72.0)		
Overweight/obese	194 (27.7)	84 (43.3)	110 (56.7)		
Depressive symptoms					
Yes	274 (39.1)	124 (45.3)	150 (54.7)	45.74 (1)	** < 0.001**
No	426 (60.9)	90 (21.1)	336 (78.9)		
Anxiety symptoms				12.23 (1)	** < 0.001**
Yes	181 (25.9)	74 (40.9)	107(59.1)		
No	519 (74.1)	140 (27.0)	379 (73.0)		
Internet addiction level				9.82 (2)	**0.007**
Severe	142 (20.3)	57 (40.1)	85 (59.9**)**		
Moderate	324 (46.3)	99 (30.6)	225 (69.4)		
No	234 (33.4)	58 (24.8)	176 (75.2)		

*BDT* Bangladeshi Taka (currency). Bolded values indicate statistically significant (p < 0.05).

### Eating disorder: prevalence and associated factors

The average score for the EAT-26 was 17.85 (SD 12.80). Based on EAT-26 screener, the prevalence of ED risk among study participants was 30.57%. The chi-square test showed participants’ gender (p = 0.008), age (p < 0.001), study level (p < 0.001), marital status (p = 0.019), monthly family income (p = 0.003), tobacco smoking status (p < 0.001), self-reported BMI status (p < 0.001), depressive symptoms (p < 0.001), anxiety symptoms (p < 0.001), and internet addiction level (p = 0.007) was significantly associated with ED (see Table 1).

Table 2 presents the findings of bivariate logistic regression analysis, showing all variables except participants’ academic performance, residence, family size, physical activity level, and sleep duration (p > 0.05) were significantly associated with the risk of ED.

**Table 2:** Bivariate logistic regression analysis showing the factors associated with the risk of disordered eating attitudes and behaviors among study participants (N = 700).

Variable	Unadjusted estimate				Overall p value using likelihood ratio test
	Odds ratio	95% CI for odds ratio		p value	
		Lower limit	Upper limit		
Gender					
Male	Reference				
Female	1.55	1.124	2.146	**0.008**	
Age in years					** < 0.001**
18–20	Reference				
21–24	1.11	0.670	1.850	0.679	
25 and above	2.25	1.293	3.915	**0.004**	
Study major					0.063
Engineering	1.44	0.806	2.561	0.291	
Health science	0.91	0.532	1.557	0.731	
Biological science	1.39	0.839	2.322	0.199	
Social science	1.83	1.023	3.308	**0.042**	
Others	Reference				
Study level					** < 0.001**
1st year	Reference				
2nd year	1.22	0.755	1.957	0.423	
3rd year	0.88	0.519	1.485	0.626	
4th year	1.40	0.839	2.338	0.197	
Post-graduation	2.80	1.649	4.723	** < 0.001**	
Academic performance					
Poor (CGPA < 3.0)	0.908	0.655	1.259	0.562	
Good (CGPA > 3.0)	Reference				
Marital status					
Single	0.607	0.399	0.925	**0.020**	
Married	Reference				
Residence					0.062
Urban	0.77	0.532	1.103	0.152	
Sub-urban	1.48	0.777	2.805	0.234	
Rural	Reference				
Family income					
≤ 35,000	Reference				
> 35,000	1.63	1.179	2.255	**0.003**	
Family size					
Nuclear (< 5 members)	0.96	0.691	1.321	0.782	
Extended (> 5members)	Reference				
Tobacco smoking status					
Yes	2.91	2.093	4.066	** < 0.001**	
No	Reference				
Physical activity level					0.934
Physically inactive	Reference				
Moderate activity	0.94	0.618	1.457	0.810	
Regular activity	1.01	0.647	1.585	0.957	
Sleep duration					0.339
Short	0.77	0.521	1.148	0.335	
Normal	Reference				
Long	0.73	0.470	1.150	0.178	
Self-reported BMI status					** < 0.001**
Underweight	0.45	0.238	0.834	**0.011**	
Normal weight	Reference				
Overweight/obese	1.97	1.377	2.803	** < 0.001**	
Depressive symptoms					
Yes	3.09	2.213	4.305	** < 0.001**	
No	Reference				
Anxiety symptoms					
Yes	1.87	1.314	2.668	** < 0.001**	
No	Reference				
Internet addiction level					**0.008**
Severe	2.04	1.300	3.185	**0.002**	
Moderate	1.34	0.914	1.951	0.135	
No	Reference				

Bolded value indicates statistically significant (p < 0.05).

The adjusted estimated effects of the factors associated with the risk of ED among study participants are shown in Table 3. The adjusted regression analysis demonstrated that female students had a higher likelihood of ED risk compared to male students (adjusted odds ratio, aOR 3.11, 95% CI 2.00 to 4.84, p < 0.001). The higher odds of having at-risk ED were found among students with depressive (aOR 3.04, 95% CI 2.69 to 6.08, p < 0.001) symptoms and anxiety (aOR 2.06, 95% CI 1.33 to 3.17, p = 0.001) symptoms compared to their counterparts. Students who had severe (aOR 3.95, 95% CI 1.92 to 8.11, p < 0.001) and moderate internet addiction (aOR 2.15, 95% CI 1.14 to 4.05, p = 0.019) were at higher odds of having at-risk ED than those who had no internet addiction (see Table 3).

**Table 3:** Multivariable logistic regression model showing the factors associated with the risk of disordered eating attitudes and behaviors among study participants (N = 700).

Variable	Adjusted estimate			
	Odds ratio	95% CI for odds ratio		p value
		Lower limit	Upper limit	
Gender				
Male	Reference			
Female	3.11	2.000	4.838	** < 0.001**
Age in years				
18–20	Reference			
21–24	1.49	0.795	2.770	0.215
25 and above	3.73	1.911	7.306	** < 0.001**
Study major				
Engineering	2.82	1.401	5.673	0.004
Health science	1.50	0.776	2.893	0.228
Biological science	1.77	0.908	3.455	0.094
Social science	3.73	1.762	7.905	**0.001**
Others	Reference			
Residence				
Urban	0.62	0.388	0.994	0.047
Sub-urban	2.34	1.026	5.327	0.043
Rural	Reference			
Family income				
≤ 35,000	Reference			
> 35,000	1.58	1.069	2.337	**0.023**
Tobacco smoking status				
Yes	3.52	2.308	5.371	** < 0.001**
No	Reference			
Physical activity level				
Physically inactive	Reference			
Moderate activity	1.58	0.808	3.095	0.181
Regular activity	2.77	1.350	5.669	**0.005**
Sleep duration				
Short	0.69	0.401	1.193	0.185
Normal	Reference			
Long	0.47	0.263	0.842	**0.011**
Self-reported BMI status				
Underweight	0.39	0.194	0.819	**0.012**
Normal weight	Reference			
Overweight/obese	3.06	1.945	4.815	** < 0.001**
Depressive symptoms				
Yes	3.04	2.685	6.084	** < 0.001**
No	Reference			
Anxiety symptoms				
Yes	2.06	1.333	3.173	**0.001**
No	Reference			
Internet addiction level				
Severe	3.95	1.924	8.106	** < 0.001**
Moderate	2.15	1.136	4.053	**0.019**
No	Reference			

Bolded value indicates statistically significant (p < 0.05).

Variables entered on full model (step 1): Gender, age, study major, study level, academic performance, marital status, residence, monthly family income, family size, tobacco smoking status, physical activity level, sleep duration, self-reported BMI, depressive symptoms, anxiety symptoms and internet addiction level.

## Discussion

The main purpose of the current study was to examine the association between mental health conditions and addictive behaviors with the risk of ED among populations in Bangladesh. Our findings show depressive symptoms, anxiety symptoms, and internet addiction were significantly associated with the risk of ED among university students in Bangladesh. These findings suggest that mental health and addictive behaviors need to be considered as part of interventions aimed at preventing ED among university students in Bangladesh.

In the present sample, the risk of ED was associated with depressive symptoms among university students. This finding is somewhat comparable with previous studies conducted among university students from ASEAN^13^ and Turkey^44,45^. Lee and Vaillancourt^46^ conducted a five-year cohort study among Canadian students (07 to 11 grades) aged between 13 and 17 years. The authors reported disordered eating behavior and depressive symptoms were significantly associated. Depression can impact students' life and can interfere with leading a healthy, active lifestyle, which may negatively affect other behaviors such as eating habits and food choices^44^. A plausible explanation linking the association between depressive symptoms and ED risk could be individuals may exhibit disordered eating behaviors to alleviate negative emotions and aversive self-awareness^47^. Further follow-up studies are recommended to identify the directionality of the association between depression and ED.

Another finding of note was the association between anxiety symptoms and ED risk. This has been reported in previous literature^45,48^. Consistent with our study, Celikel et al. revealed that anxiety symptoms were significantly associated with disordered eating attitudes among college students in Turkey^45^. Students may be more prone to mental health challenges due to the pressures of academic achievement and managing busy daily schedules. Studies have shown that increased anxiety levels in students are related to higher levels of stress and emotion, which could manifest in less-desirable behaviors such as disordered eating^49^. Future studies that explore potential moderators or mediators of the observed relationships (i.e., mental health conditions with the risk of ED) are highly suggested.

Results from our study showed students classified as having moderate to severe internet addiction were more likely to have an ED risk compared to their non-addicted counterparts. Internet addiction among university students in Bangladesh is substantially high (68.4%)^42^. This is not surprising given the ease by which students can access the internet via smartphone or computer at any time of the day. These high levels of internet usage play a significant role in internet addiction and may be influencing students’ eating patterns and habits^50^. The evidence of an association between internet addiction and disordered eating attitude/ED has been reported in numerous studies^51,52^. For instance, a previous Turkish study demonstrated that university students’ susceptibility to eating behavior disorders increased with their addiction to internet^51^. Although internet facilities have made people's lives easier to varying degrees, excessive use of the internet may predispose young folks to adopt unrealistic body ideals of celebrities as role models, leading to the onset of EDs such as anorexia and bulimia nervosa^30^. In addition, frequent internet use increases the likelihood of online food ordering (typically fast food and high-caloric food), which may increase the chance of developing unhealthy behaviors such as overeating and binge eating disorder. Further longitudinal studies are recommended to explore the relationship between internet addiction and different forms of eating pathologies, such as restrictive eating disorder (i.e., dieting) and binge eating disorder. It is imperative that public health practitioners are aware of the role that increased internet usage can play in shaping health behaviors and outcomes. Adopting university-wide awareness campaigns on the potential harm increased internet usage may have on students’ health is needed.

In Bangladesh, the prevalence of ED is rising substantially among university students^16^. According to the present study, around one-third of the students showed ED risk. This prevalence was higher than a previous study conducted among university students in ASEAN countries, with reported prevalence ranging from 6.8% in Thailand to 20.6% in Myanmar^13^. This alarming trend of the prevalence of ED risk among Bangladeshi universities recommends the need for evidence-based guidelines that are guided and implemented by qualified personnel to help with the assessment, diagnosis, and treatment of EDs. Several other sociodemographic and behavioral factors such as gender, BMI status, tobacco smoking habits and study discipline (i.e., major) were significantly associated with the risk of ED among study participants. Previous studies have also reported similar findings^15–17,53^.

The findings of this study also support our hypothesis that female students were more likely to develop an ED risk than male students. This result is similar to previous studies carried out among university students in Bangladesh^17^, Malaysia^54^, and Turkey^55^. This observed relationship could be explained by studies that have found females have higher body dissatisfaction and place greater importance on physical appearance^56^, which may predispose them to develop ED risk or unfavorable eating behaviors. Further, it is plausible that internal motivating factors, such as a desire to lose weight to get an ideal body shape, made them vulnerable to developing unhealthy eating habits^54^. Lastly, females in Bangladesh seek to adopt a modern culture that might be associated with ED risk. For example, a recent Bangladeshi study showed that young female university students with a strong interest in modern culture (such as fashionable clothes and Indian TV shows) had a higher likelihood of developing an ED risk^18^. Further qualitative studies are recommended to better understand the underlying motivating factors that place Bangladeshi female students at a greater at-risk of having ED than male students.

Generally, a co-occurrence of ED with mental health distress, such as depression and anxiety, may lead to more serious health conditions, and in some cases, death^27^. Therefore, a concerted effort to establish campus resources and /or programs involving multiple parties such as university administrators, public health policymakers, mental health professionals, and nutritionists is needed. For example, setting up a "Mental Health Center" on university campuses for students to receive counseling (such as cognitive-behavioral therapy) to address any mental health-related issues could be one strategy. Cognitive-behavioral therapy is a well-established and effective approach for treating EDs, which focuses on identifying, understanding, and changing thinking and behavior patterns^57^.

### Strengths and limitations

This study had several strengths. It was one of the first studies to explore how mental health conditions and addictive behaviors are associated with the risk of ED among university students in Bangladesh. Being a baseline evidence for Bangladesh, these findings would assist university authorities and policymakers to develop behavioral interventions and coping strategies to reduce the risk of ED. Another strength of this study is its large sample size, validated screening tool, and robust methodological approaches. This study was not without limitations. Causal interference between outcome variable and explanatory variables is limited due to the cross-sectional study design. We did not perform any clinical examination for diagnosing EDs, and an ED cannot be diagnosed reliably with a single screening technique^43^. Since the EAT-26 represents restrictive eating pathology and does not precisely detect binge eating^58^, our study findings could not estimate the real picture of binge eating in this subject group. Furthermore, the EAT-26 has been questioned for its utility in estimating ED risk in individuals at low-risk for ED (for example; overweight and obese individuals undergoing weight loss treatment)^59^. Lastly, due to the self-report nature of our measures, response bias and social-desirability may be present in our current data.

## Conclusion

In our study sample, approximately three in ten students reported at-risk ED. This study showed, among university students in Bangladesh, a higher risk of ED was found among students with internet addiction, as well as students reporting higher levels of depressive and anxiety symptoms. These findings will guide policy experts, public health practitioners, and university authorities to adopt potential strategies (e.g., implementing mental health awareness programs and resources on university campuses in Bangladesh) emphasizing students' mental well-being and addictive behaviors to reduce the risk of ED. Future experimental or longitudinal studies in university students are recommended to gain a better understanding about causal factors of ED to support intervention initiatives and strategies by public health practitioners and policy experts.

## Data Availability

The datasets and questionnaire used and/or analyzed during the current study will be shared upon request to the corresponding author.
